# “I represent the police I represent the state” – Justification work following ethno-national boundaries crossing among Arab female police officers in Israel

**DOI:** 10.3389/fsoc.2023.1296790

**Published:** 2023-12-20

**Authors:** Tal Meler

**Affiliations:** Zefat Academic College, Safed, Israel

**Keywords:** boundaries crossing, Arab female police officers, justification work, indigenous women, ethno-national identity

## Abstract

**Introduction:**

In recent decades, Israel’s public sector diversity policy has led to the recruitment of many Arab female police officers (FAPO). For Arab women, joining the police force is seen as boundary-crossing, highlighting the tension between their professional, civilian, and ethno-national identities. While they are Israeli citizens, Arabs are often perceived as an unassimilated minority due to nationality, religion and culture. Arab women face numerous obstacles in integrating into the broader labor market, both from the state and their own society. Therefore, entry into the public sector, like the police force, is of great significance. However, some may view joining the Israeli police as cooperating with the majority-hegemonic group or even as betrayal.

**Method:**

Qualitative, semi-structured interviews were conducted with 27 FAPO employed by the Israel Police asking how they articulate the ethno-national identity complexity in their pursuit of quality jobs in the Israeli police force, and how they justify dealing with the challenges this presents.

**Results:**

The analysis sheds light on the interviewees’ subjective perspective of their recruitment to the police force, i.e., the ways they experience their crossing of ethno-national boundaries and the conflictual identity experience inherent in the process.

**Discussion:**

These insights offer a better understanding of the FAPO’s experiences regarding their justification work while facing criticism and value conflicts. The contributions of this study are threefold: 1. It advances the literature on the labor market integration of Arab women from the theoretical perspective of boundary-crossing. 2. It adds to the theory of boundary-crossing in the labor market for minority women in distinct locations. 3. It provides insights into the subjective perspectives and experiences of FAPO, contributing to organizational knowledge about minority policing in a deeply divided society characterized by tense relations between the minority and the police.

## Introduction

Since the 1960s, police diversity has been suggested as a means of improving police-citizen relationships, particularly in minority communities (Todaka et al., 2018). In general, diversity in organizations may be perceived as implementing the strengths of racialized minority groups, and may help in dealing with exclusionary mechanisms applied to the social ‘other’. Based on the notion of representative bureaucracy, a police department representing the demographics of the population it serves should produce measurable improvements in policing and crime-related outcomes, both directly and indirectly (Lim, 2006). However, recruiting minorities into policing, and in particular women from minority groups, reveals the multi-layered complexity of the intersection of gender and ethno-national identities with diversity promotion projects.

The police department in Israel, as elsewhere, faces varied challenges in its attempts to increase diversity. Israel is a diverse society whose demographics fall under the definition of ‘super diverse’. As such, it serves as an example of a conflictual society with tense relationships between majority and minority groups. Moreover, there are significant gaps between the two social categories: the Jewish citizens who constitute the majority group and the Arab^1^ citizens who constitute the minority group. These gaps exist regarding resource distribution, ideological perceptions, identity, culture, policy preferences, definition of state, and other issues (Smooha, 2020). The more that a society is regarded as conflictual, the more central the role of the conflict is in the relationships among the groups, and the more severe the conflict (Smooha, 2020).

One of the prominent issues affected by the conflict is the integration of Arab women into the labor market. Like minorities elsewhere, Arab women are the first to be hit by a recession, the first to be ejected from the workplace, and the last to reap the fruits of economic growth. Data concerning Arab women’s integration into the Israeli labor market indicates its segmentation. The dearth of job opportunities in the periphery worsen this situation, further marginalizing Arabs in the job market (Khattab and Miaari, 2013). Furthermore, even when Arab women integrate into the labor market, this is a limited integration only, highlighting not only the scarcity of opportunities and structural barriers but also the impact of social constraints compelling women to seek employment within their local communities.

Anthias (2008, 2020) introduced the theoretical concept of ‘crossing boundaries’ as crucial to understanding women’s positioning and their endeavor to broaden the opportunities accessible to them. In her analysis of these processes, she refers to spaces that offer belonging as opposed to spaces that project non-belonging (Anthias, 2008). For minority women in underdeveloped regions with limited labor market opportunities, crossing ethno-national boundaries becomes a crucial necessity. However, the tense political-social context colors the entire process of boundary-crossing and diversity policy, and is the basis for understanding the issue discussed in the current article. Arab women who respond to the opportunity offered by the Israeli Police as part of a diversity policy need to cross the symbolic and social-national boundaries between their society and the hegemonic society, and therefore transgress the collective norms of both. Within the loaded context of majority-minority relations, they challenge the social order and collective identities mainly based on gender and ethno-nationality. This experience echoes the research of Oldenhof et al. (2014), who examined civil servants’ value preferences and conflicts. For Arab women, serving in the Israeli police means facing criticism and value conflicts, which involve, according to Oldenhof et al. (2014, p. 60) “continuous justification work, which includes rebuilding existing compromises, creating new compromises, and justifying these to significant others.”

Therefore, the case of female Arab police officers (FAPO) serving in the Israeli police force deals not only with police diversity, also emphasizing the contradictions between their professional identity and their civilian and national identities.^2^ The recruitment of Arab women into the Israeli police constitutes a significant opportunity for understanding the theory of crossing boundaries - an ongoing process where women are recruited to jobs beyond the conventional opportunities available to their minority (Anthias, 2008, 2020; Meler and Benjamin, 2022). For some members of their communities, it is less expected that Arab women would be employed by the Israeli police, perceived as oppressive toward members of their minority.

Using the justification framework of Boltanski and Thévenot (2000), and following Oldenhof et al. (2014), enabled me to look at the value choices and preferences, i.e., ‘justification work’, of indigenous female police officers, while emphasizing the importance of taking into account the social context of the police officers when explaining their values and actions. Therefore, the Israeli situation challenges prevailing theoretical discussions of boundary-crossing linked to diversity policies (Linnehan and Konrad, 1999), and efforts to enhance minority group representation in representative bureaucracies (Bradbury and Kellough, 2011). These discussions usually center on scenarios in which the minority group seeks inclusion within public sector institutions. The present study may provide a better understanding of the complex relationship between the minority and the majority, in which the high level of tension raises the possibility that some members of the minority group are not interested in such representation and may therefore change the meaning of the employment paths that the diversity policy opens up. This gap in the literature raises the need to examine the meaning of these unique occupational paths, and even more so from a gender perspective, focusing on the occupational experience of minority women crossing the ethno-national boundaries, emphasizing the way in which they themselves experience the disapproval of their communities regarding their following these occupational paths. Moreover, in this article I begin to fill in the gaps in the research of Arab women’s employment, regarding the conflicting values they may experience in mixed work spaces by focusing on compromises and justification work.

The study goes beyond the prevalent assumptions regarding women’s cooperation with their communities, which do not take into account the range of the crossing boundaries processes which women undertake in order to enhance access to resources. Using semi-structured in-depth interviews with FAPO in Israel, I asked how do Arab native minority women articulate for themselves the ethno-national-identity complexity involved in their attempt to obtain a quality job in the Israeli police force, and what are the justifications they adopt in order to re-formulate what may be considered as crossing ethno-national boundaries and even a betrayal among some of their family or community members. The integration of minority women into a diverse organization may exposes identity conflicts, even with colleagues from the majority group (Farrell and Barao, 2023). However, this article focuses on the internal dynamics within the community and the voices that the FAPO express toward their community members. So far, little attention has been paid to the subjective perspective of Arab women on their coping mechanisms with simultaneous conflicting values embedded in their crossing of boundaries.

The contributions of this study are threefold: 1. It contributes to the literature regarding the labor market integration of Arab women from the theoretical perspective of boundary-crossing, focusing on their justification work. 2. It adds to the theory of boundary-crossing in the labor market for minority women in distinct locations, analyzing how they reconcile justifications in order to deal with conflicting values. 3. It provides insights into the subjective perspectives and experiences of FAPO, contributing to organizational knowledge about minority policing in a deeply divided society characterized by tense relations between the minority and the police.

Below I develop one aspect of the theory of boundary-crossing, enabling me to address Arab women’s subjective perspectives toward the political and ethno-national tensions embodied in their recruitment into the police department; introducing existing knowledge regarding the national identity of Arabs in Israel, their status (particularly education and employment), and the relationship between the police and the Arab population in Israel. All of these provide the background which clarifies the ethno-national complexity of crossing boundaries in the integration of Arab women, minority women in an ethnic democracy, into the police force.

## Literature review

### Boundary crossing of minority women

The concept of boundary-crossing theory was initially introduced by Anthias (2008, 2020), who highlighted the significance of belonging dynamics. Her idea that community members can uphold their sense of belonging while rejecting certain associated values highlights the ongoing negotiation inherent in boundary-crossing. Previous studies have deepened our comprehension of this dynamic process by emphasizing the initiative of individuals who cross gender boundaries and examining workplace resistance among women who challenge hierarchical gender-based organizational boundaries (Jamjoom and Mills, 2022). These authors, who demonstrated how women cross the gender boundaries set by organizational hierarchies, present alternative interpretations of their actions in their fight against discrimination and marginalization. This strengthened the claim that crossing ethno-national/gender boundaries is an ongoing process (e.g., Anthias, 2008, 2020). Similarly, more recently Wasserman and Frenkel (2020) conducted a case study on Ultra-religious Jewish women working in Israeli high-tech companies. This study can also be viewed as dealing with the crossing of gender boundaries. Their research highlights how the intertwining of gender and religiosity exposes these women to conflicting visibility expectations from managers, peers and their own religious community.

Integrating women from these backgrounds into organizations is challenging, as their communities employ surveillance mechanisms to enforce strict chastity rules and discourage work in mixed-gender settings. At the same time, the workplace subjects them to other surveillance methods aimed at ensuring their alignment with the modern ideal of a dedicated employee who is socially integrated with colleagues and embodies the organization’s values (Acker, 1990). However, there is currently a knowledge gap regarding how minority women who have successfully crossed boundaries to attain higher-quality jobs beyond traditional opportunities cope with their communities’ disapproval and devaluation of their work. This challenge goes beyond only gender perspectives, it is also influenced by their ethno-national background. Women in this situation must navigate the dissonance between their actions and the national and conservative expectations of their families and communities. For women whose employment faces objections based on ethno-nationality rather than just gender, this framework offers valuable insights into the hurdles they must overcome and their resistance to societal condemnation. Addressing this gap is crucial for the development of a boundary-crossing theory which recognizes its ongoing, evolving nature. In this process, minority women strive to expand their spheres of belonging (Anthias, 2008, 2020) and enhance their access to resources, while resisting societal efforts to restrict their sense of belonging and control over resources. As I show in the present article, boundary crossing is not only the connection to the labor force and the extraction from the ethno-national enclave and the offensive workspace, it is also the process where the gender labor space is undermined, new labor domains are formed, women enter new economic-political areas, and accumulate power and influence in the community. Moreover, the recruitment of the FAPO will reveal cracks in the rigid ethno-national-religious boundaries which prevail among the social groups in Israel. This may promote perceptions of civil nationalism based on cooperation on the basis of mutual respect (Ignatieff, 1993), and establish belonging in circles crossing the boundaries of the ethno-national community. These processes also affect family power relations and gender relations, just like the barriers women had to deal with when they integrated into the public sphere. Women from minority groups are faced with emerging value conflicts, and have to cope with various mechanisms and justifications to make sense of their often contradictory and tension-ridden multiple subject positions in the process of boundary crossing (Boltanski and Thévenot, 2000; Oldenhof et al., 2014).

Therefore, exposing the subjective perspective of FAPO’s experience enables me to broaden the theoretical understanding of the ethno-national layer of boundary crossing by focusing on their experience and the ways in which minority women constitute their perspectives and justification work under the condition of the communities’ objection to their employment.

### Nationalism and national identity design in Israel

Post-colonialist theory presents a more nuanced view of identity than the traditional dichotomous one. Identity is recognized as multifaceted, encompassing dimensions such as gender, ethno-nationality, religion and culture. The fluid nature of identity means it can change within different social contexts, even concurrently, especially when adopting a hybrid identity (Arweck and Nesbitt, 2010). In general, individuals’ identities are shaped by the influence of their upbringing and educational environments. Heritage, culture, values and language are acquired both directly and indirectly through society, family, and education (Ben-Asher et al., 2023).

The formation of an identity expresses two conflicting needs: maintaining personal uniqueness and belonging to a group (Brewer, 1991). Thus, every conception or understanding of the self is connected with a person’s cultural environment, including its characteristics and values (Bruner, 1990), and is dependent on one’s group affiliation (Tajfel and Turner, 1986). Consequently, although the definition of identity originates in a conception of the self within a certain context and time frame (Beijaard et al., 2004), development of the self is an ongoing process of reciprocal relations between the individual and the environment, hence changes in its definition are likely to take place throughout one’s life. The development of a bicultural identity is a key process during adolescence, which persists throughout one’s life and is periodically reconsidered in response to significant life events, such as education and professional achievements (Sodhi, 2008).

Israel is defined as a Jewish democratic state. Arabs, who are Israeli citizens, constitute a minority national group, comprising around 21% of the population (Israel Central Bureau of Statistics, 2023). Although born in Israel, Arabs are considered an unassimilated minority, differentiated by nationality, religion, culture and language. Most Jews and Arabs in Israel live in separate municipal localities with separate education systems, political parties, and media outlets (Smooha, 2020). Furthermore, the cultural-political context of Arab civil society in Israel is particularly complex. As a minority they suffer discrimination, exclusion and oppression by Jewish Israeli society, and are deeply divided from it (Smooha, 2002).

As a result of the civil exclusion, according to Jamal (2007), the citizenship of Arabs in Israel can be defined as a ‘hollow citizenship’. This concept refers to the fact that citizens who are entitled by law to equal rights have to deal with a low level of commitment by the state. This stems from the discourse of exclusion, and includes processes taking place in three circles: political, economic and social, which feed into each other and render formal citizenship meaningless. Moreover, the Israeli context, constituted around Zionist-Jewish cultural history and ideology, operates according to practices and values foreign to Arab society and even antagonistic to it. Israeli political culture is focused on the values of Zionism, which strives to establish the status of the Jewish people in the State of Israel and promote their control over its political space (Jamal et al., 2019). These practices were further intensified after the passing of the Nation-State Bill of 2018, which exacerbated the discrimination on the basis of nationality in the allocation of land and resources, and strengthened the exclusion of Israel’s Arab citizens.

In Israel, there is a lack of a common civil nationalism shared by all citizens based on common citizenship and territory (Smooha, 2002, 2006). Both Jewish and Arab citizens are influenced by collectivist orientations, with distinct identities, loyalties and strong national interests that continue to create divisions. Zionist ideology, predominant among Israeli Jews, serves as a mechanism of separation and exclusion concerning the country’s Arab citizens. There is a consensus that ethno-nationality governs social life in Israel, excluding non-Jewish groups from significant aspects and distancing them from the core values of Israeli society. This principle elevates Zionist ideology, granting privileges to Jews while placing Arab citizens on the outskirts of Israeli society (Jamal, 2007; Bauml, 2010). While the state of Israel provides certain rights to all citizens, it also allocates land, economic resources and job opportunities based on criteria rooted in Zionist priorities, Jewishness, or fulfillment of military service, from which Arabs are legally exempt. Consequently, the discourse of Israeli citizenship is seen as hierarchical and deferential, establishing inequalities between those perceived as contributing more or less to the community (Shafir and Peled, 2002; Peled, 2008).

For the past several decades there has been an ongoing discussion of the identity of the Arab minority in Israel, with an emphasis on the complexity of this identity and examination of the different identity placements (such as cultural identity, national belonging, citizenship, a place of living, belonging to a minority group, religious belonging and gender-related belonging). The ethno-national conflicts between Jews and Arabs in Israel have important implications for understanding their identity processes (Smooha, 2020).

Fakhoury (2021) discusses the historical and cultural perception that Arab-Palestinians are part of the Arab-Palestinian national identity, despite the growing influence of Israelization since the 1990s. He argues that, culturally, labeling a person as an Arab-Israeli does not substantially alter their life or distinguish them from Arab-Palestinians, as they remain deeply connected to Arab-Palestinian culture. Fakhoury (2021) further adds that, in terms of lived experiences, Arab-Israelis face similar discrimination to Arab-Palestinians. Israeli politics, driven by a national agenda, discriminates against Arabs based on their origin and religion, regardless of their self-identification.

### Arab society: a social, civil, political survey

In many dimensions of social stratification, due to their civil status as well as social and cultural perceptions, Arabs in Israel suffer from inequality and oppression in many aspects of life in comparison with the Jewish majority, including the economy, employment, politics and education (Gharrah, 2018). Thus, Smooha (2020) believes that concerning the different social categories in Israeli society, Arab are the group suffering from the deepest divide. Some scholars contend that in this respect, the state of Israel, like other colonizing countries, consciously engages in policies typified by ‘supervised abandonment’ (Bauml, 2010), based on many years of discrimination, exclusion and neglect, despite the inclusion of Arabs in Israel’s formal civic frameworks.

However, although the separation between the two ethno-national groups is fundamental and carries crucial implications, at the same time, in recent decades the Arab minority has gradually integrated into the dominant Israeli culture, assuming a growing role in mainstream institutions. More specifically, in the past decade, the number of Arabs in Israel with an academic education has increased, particularly women.

Side by side with these social changes, there is a clear rise in the value of individualism in society, as well as a rise in the global discourse regarding native minority rights and human rights and the strengthening of a middle class (Jamal et al., 2019). The strengthening of civil society among the Arab population, the rise in political awareness among them, the involvement of young people in social initiatives, and the success of Arab social organizations in making social changes, demonstrate a social reality offering an alternative to government organizations or complementing state-provided services with alternative civil organizations. In addition, these patterns cause trends of autonomous development on the one hand, but strengthen traditional, patriarchal inter-community forces on the other (Jamal et al., 2019).

The population of young Arabs in Israel (18–35) is approximately 22% of the general Israeli population of the same age range (Granit, 2021). These young people deal with the familiar issues all young people in Israel and worldwide deal with, such as integrating into the academic system and the labor market, career development, starting a family and self-realization. Simultaneously, they are also busy with additional needs and issues stemming from their belonging to a minority group and the political circumstances accompanying this. Thus, their identity issue often suffers from a clash between the Western set of values usually represented by Jewish-Israeli society values, and a more conservative set of values. This clash causes internal tension among young Arab people, who are constantly dealing with the move between two tracks – conservatism and religiosity on the one hand and Westernism on the other (Rudich-Cohen and Sheperman, 2015; Granit, 2021).

The issue of self-identification as a national minority is characterized by a sense of disappointment – many of them feel that Israel oppresses them, does not allocate sufficient resources to their society, and provides them with few opportunities for mobilizing their inner resources. These feelings are accompanied by inequality in the resources allocated to the Arab minority and fewer voluntary public responses. These differences in resource allocation stem from the fact that most public resources for young people in Israel are aimed, as mentioned, at those who served in the IDF or its alternative, the National Service, and are therefore eligible by law for different benefits. As a result, it seems that young Arabs in Israel are discriminated against, excluded from decision-making processes, and suffer from high rates of socio-economic marginalization (Rudich-Cohen and Sheperman, 2015; Miaari and Hadad Haj-Yahya, 2017).

Indeed, the latest data (Miaari and Hadad Haj-Yahya, 2017) show that the rate of NEET (Not in Education, Employment or Training) or idleness as a state of not belonging to any setting (workplace or academic) among young people in Israel aged 15 to 29 is 28% - one of the highest in the OECD. Idleness is particularly common among Arabs (37%). These data reflect the obstacles that young Arabs in Israel face during the stage in life when they are supposed to enter the job market or begin their studies. This is a result of the fact that they are members of an ethno-national minority, and their reality is characterized by limited opportunities, discrimination in many spheres, living in peripheral regions, and little access to public transportation between Arab residential communities and employment zones (Miaari and Hadad Haj-Yahya, 2017). From a gender perspective, these issues take on a more serious meaning in relation to women’s integration into the labor market.

### Arab women: integration into the general Israeli labor market

Various studies have discussed the complexity characterizing the daily lives of Arab women in Israel, who experience multiple realities simultaneously. They live their lives in spaces demanding mastery of a language of mixed cultures and imposing ambivalent, ‘hyphenated’ realities in terms of identity and independence. Under such conditions, oppression and self-preservation are interwoven with challenges and resistance (Abu-Rabia-Queder, 2017; Meler, 2019; Sa'ar, 2023). Despite exclusion from the Israeli hegemony and the multiple economic, status-related marginality and political tensions that shape their lives, they have been affected by recent processes taking place in Israeli society. Over the past three decades, various changes have been reshaping the options available to them in numerous aspects of life. These changes include increased access to higher education, greater participation in the labor market, and changes in traditional-patriarchal perceptions (Abu-Rabia-Queder, 2017; Meler, 2017; Sa'ar, 2023).

However, despite the growing number of employed educated Arab women, many are still subjected to the double burden created by social exclusion, under-development of minority populated residential areas and the control mechanisms of both the state and Arab society (Meler, 2019). As a result, Arab women suffer from high rates of unemployment, underemployment, part-time employment or employment saturation in teaching jobs (Meler, 2019). Therefore, in this narrow structure of employment opportunities, quality jobs in the public sector constitute a particularly salient component (Meler and Benjamin, 2022).

Sa'ar and Younis (2021) note that Arab women integrating into the general Israeli labor market have to deal with two scripts. The first is the gender script rooted in the gendered patriarchal contract, shaping women’s roles and daily lives in both private and public spheres, including their inclusion in the labor market. The second script involves Jewish dominance, associating success, leadership, numerical superiority and higher salaries with the majority group. Scripts have an impact on the opportunities open to women and their ability to act in real life situations. Nevertheless, even as they integrate into more privileged echelons of the labor market, allowing those with higher education to expand their civil rights beyond nominal levels, nationality continues to impose clear limitations on the extent and nature of their participation (Sa'ar, 2023).

Similarly, Abu-Rabia-Queder (2017) points at the complexities’ experienced by professional women from a minority group when they have to maneuver between racialization and patriarchy. Dealing with many layers of oppression structures women’s daily lives through social divisions of power. Barriers of prejudice and discrimination are inherent in the Israeli labor market. Against the background of the national conflict, racialization occurs in the employment space, even if the ethos of neutrality seemingly shapes the character of organizations in Israel as being egalitarian, non-political spaces. Despite the official stance, there are still various manifestations of tension between Jewish and Arabs professionals in mixed organizations, not all of which are addressed (Abu-Rabia-Queder, 2017; Popper-Giveon and Keshet, 2020; Sa'ar and Younis, 2021; Meler and Benjamin, 2022).

Indeed, in the last decade there is a growing trend of Arab women who have chosen to cross spatial and ethno-national barriers in pursuit of employment in their field of training (Abu-Rabia-Queder, 2017; Popper-Giveon and Keshet, 2020; Meler, 2021; Sa'ar and Younis, 2021; Meler and Benjamin, 2022). Negotiating the crossing of boundaries improves their chances of better integrating into quality jobs in the public sector in mixed workplaces, thus they insist on claiming their belonging and their rights, despite signs of non-belonging (Abu-Rabia-Queder, 2017; Popper-Giveon and Keshet, 2020; Meler, 2021; Sa'ar and Younis, 2021; Meler and Benjamin, 2022).

These trends do not undermine the institutional perspective which highlights exclusion of the minority, rather adding a dimension of action. Action is enabled when the spatial map of opportunities, combined with accessibility of interpersonal and material resources, encourages the crossing of boundaries (Meler and Benjamin, 2022). This provision of a broader opportunity structure was generated, for example, by the introduction of two policies to the Israeli public sector: the first is a diversity policy opening senior public service administrative positions to Arab women; and the second is a representative bureaucracy policy, calling for more minority women in public service in an effort to improve majority-minority relationships and trust. However, prior research has concentrated on integration within mixed work environments, such as the education, welfare, health, law, or high-tech sectors, where women may garner respect and recognition. It is crucial to comprehend the mechanisms of boundary crossing within contentious organizations which hold political significance and confront women’s ethno-national or civic identity. Employment as police officers for Arab female citizens in Israel may be questioning their values and signifying them as inappropriate members in their communities. Such employment may require the making of daily compromises and justifications to significant others. Following Oldenhof et al. (2014), because compromises are fragile and open to criticism, workers have to perform continuous ‘justification work’ that entails not only the use of rhetoric but also the adaption of behavior and material objects.

### The relationship between the police and the Arab population in Israel

In Israel, as in many other Western countries, the multicultural reality, which includes ethno-national, religious and linguistic diversity, poses many challenges and dilemmas for police organizations. Violent clashes between police and minorities in many countries including liberal democracies, as well as allegations of discrimination, indicate the difficulty of the state and the police in dealing with these challenges (Hasisi and Weitzer, 2007). The charged relations between police and minorities exist within a broader context of majority-minority relations and minority relations with the state, and are also derived from economic and social problems not directly related to police work (Hinton, 2006; Hasisi and Litmanovitz, 2021).

In this context, there is a constant crisis of trust between Israel’s Arab citizens and the police, accompanied by feelings of alienation, hostility and resentment when the organizing principle of these relations is the construction of their public image as dangerous in the militaristic-security sense (Hasisi and Litmanovitz, 2021).

Diversity policy is one of the main solutions for enhancing trust between the state and the minority. In addition, there is also the need to increase cultural awareness and address the unique needs of Arab society, while trying to expand police services and make them accessible in various localities. According to the contact theory (Dhont et al., 2010), enlistment of minorities into the police force may change it from within by lowering the level of negative stereotypes toward minority groups, changing the organizational culture and improving treatment of minorities. In general, the possibility of improving the organization’s image in the public eye in general, and for the minority group specifically, is attributed to the presence of minorities in the police force. The relationships between minority groups and the police force in divided societies are influenced by the manner in which they evaluate the minority citizens’ loyalty to or alienation from the state (Hasisi and Litmanovitz, 2021).

In recent years, as part of an attempt by the Israeli police department to improve its relations with minority groups, as well as its service and appearance, it has been implementing a policy promoting diversity in the police force, i.e., increasing the representation of minority groups. Up to date data show the mobilization of more than 150 Arab women (Muslim, Christian, Druze and Bedouin) into the police force. However, in the context of the religious conservative communities of national native minorities, taking up the offered employment opportunity constitutes a boundary crossing and triggers community objection (Meler and Benjamin, 2023). In the case of Arab women enlisting to the Israeli police, this profession is not perceived as suitable for them, not only in a traditional patriarchal context, but also in the national context, as it is seen as cooperation with the dominant group.

## Materials and methods

This article is based on qualitative research following feminist semi-structured in-depth interview studies (e.g., Reinharz, 1992), which is well suited for examining the subjective perspective of women in their own words.

### Participants

This article is part of a broader research project on the integration of Arab women (with and without academic education) into the Israeli labor market, and includes Arab women who responded to the diversity policy enacted by the Israeli police.

A little over 150 FAPO currently serve in the Israeli police, belonging to the four religious/ethnic groups (Muslim/Christian/Druze/Bedouin) in Israel, and to various social statuses (education/marital status/motherhood). 16 have been employed by the police for over 15 years, 33 have been there between 5 and 15 years, and 115 have been employed for less than 5 years. Some investigate crimes and domestic violence, while others work in intelligence or in patrol teams. I interviewed 27 women in total. Participants were aged 24–45; three are divorced, 11 are married, and 13 are single. 12 have children of diverse ages. Four hold an M.A., 20 hold a B.A. degree, and three have a secondary education.

### Research tools and data analysis method

Beyond the approval of institutional ethics committees to conduct the study, it is not possible to interview police officers in Israel without formal permission. From the moment I contacted the Israeli police, obtaining permission involved a multi-step bureaucratic process that lasted several years, until receiving official permission to conduct the study.^3^ However, when this was obtained, the contact information for all currently employed FAPO was provided. I contacted potential participants by phone, provided them with an explanation of the study, and assured them that their participation was voluntary, confidentiality was ensured, and their anonymity complete. A few refused to participate in the study but the majority agreed willingly. Despite their enthusiasm for the topic, time constraints often limited their availability for interviews.

I conducted the interviews in 2022 at the interviewees’ respective police stations, scheduled at their convenience to ensure privacy. Conducted in Hebrew, each interview lasted approximately 90 min and was recorded with the interviewees’ consent. Following feminist research principles aimed at minimizing power relations in the interviews (Reinharz, 1992), participants were encouraged to express themselves freely, and relevant topics were discussed. As Edwards (1993) notes, the interviewer can affect interviewees’ narratives, reactions and behavior, creating a “double subjectivity” when subject matter is sensitive. However, encounters between researcher and interviewees also bring ethno-nationality, status and educational differences to the foreground. Although I am a woman, I have had to grapple with my positioning as a middle-class, secular Jewish Ashkenazi Israeli woman researcher. Thus, I am a privileged woman in Israeli society, and interviewing Arab women who are subordinated in Israel further complicates discussions of their private lives. This may raise a question of whether researchers such as myself are even entitled to write about Arab women (e.g., Abdo and Lentin, 2002). However, this study of FAPO, conducted in police stations, challenged me beyond the usual research power relations issue, due to my position as a feminist activist citizen vis-à-vis representatives of law enforcement. Moreover, it is important to note that from the interviews it emerged that FAPO usually feel that due to the workload and greedy institution, they are not listened to by the organization, and they felt that the interview was a unique situation for them to raise their voice and unfold their daily difficulties, created by their positive response to the diversity policy.

Additionally, because FAPO are seen by their communities as collaborating with an oppressive institution, they do not usually feel sufficiently supported to express their feelings. Thus, speaking to an ‘outsider’ seemed like a good opportunity to be open and direct about the barriers which they experience. During the interviews with these women, I immersed myself in the diverse range of experiences they were willing to recount. I was particularly focused on comprehending their descriptions. I posed open-ended questions regarding their recruitment, professional paths of crossing boundaries that were also integrated with their personal lives, reasons for joining the police force, experiences of assimilation within the organization, identities, and their family and community’s stance regarding their careers. I was constantly amazed at the intriguing directions each question led our conversations. Remarkably, the FAPO were exceptionally candid, sharing intimate details of their personal journeys.

To protect anonymity, pseudonyms were assigned to all participants, and identifying information was altered without compromising the interpretation of the findings, such as years of seniority, work location or position. The recorded interviews were analyzed thematically (Charmaz, 2015) in three stages. First, all the data were read in order to become familiar with their contents and understand their potential to contribute to a comprehensive picture of the theoretical framework of crossing-boundaries, and to ensure that they were complete. This stage required extreme sensitivity and many repetitions of interview transcripts. Second, the data were sorted with the help of significance tests and connections were identified among the texts. At the third stage, the themes were collected under primary and secondary headers (distinguishing among different paths of crossing-boundaries and focusing on the ethno-national aspect) to elicit answers to the research questions from the items of information and identified connections among the data.

## Results

Recruiting Arab women to the Israeli Police is, as mentioned, a process involving the crossing of gender, ethno-national and spatial boundaries. Here, I present the analysis of interviewees’ subjective perspective of their recruitment to the police force, i.e., the ways they experience their crossing of ethno-national boundaries and the conflictual identity experience inherent in the process.

### Training course for female police officers: the first baptism by fire

Once recruits pass their initial physical test, oral interviews, written tests and psychological exams upon entry into the organization, those who are recruited have to enter the police academy, which takes 4 to 6 months to complete and involves both classroom-centered and physical components. Recruits study the law, investigations, combat, firearms, tactical driving, computer techniques and other skills necessary to become police officers (e.g., Green, 2021). This is usually the initial encounter with the organization. In Israel, as elsewhere, the course is conducted under boarding conditions and in a military atmosphere. Arab women who are exempt from military service arrive at the police academy with no former experience in such things; and even beyond that, for some of them as girls from a conservative society, with no experience living outside their homes. The course is a kind of ‘baptism by fire’ for the future in their path of crossing spatial, national and gender boundaries, with the complexity of integration into a mixed gender and ethno-national social framework, conducted all in Hebrew, also intertwined. And this is how Lubna describes it:

*In the course I had adjustment difficulties, it was very difficult for me to leave home for a new place, and it was also the first time I had left home and it was a great distance, and I was the only Arab in the course, and sometimes there were cases where I had to fight the Jews because I am an Arab. Some of the Jews do not accept us as Arabs and hate us. With God’s help, I was [eventually] one of the leading women in the course, I was a candidate for the best trainee award, and still there is the difficulty of being alone*… (29, single, Muslim).

Young Arab women face more barriers than men in integrating into the higher education system or labor market. As young women, they are even less exposed to Jewish culture and to a mixed (gender/ethnic) environment (Meler, 2021). Similarly, from Lubna’s words we learn about her dealing with the crossing of ethno-national and spatial boundaries, and how this manifested in practice when she left home for the first time for a course with norms foreign to Arab women, and with a schedule intensive in many respects: social, physical, intellectual and personal-emotional. Lubna had an experience which sounds empowering on the one hand, in light of her having to cope for the first time and the fact that she perceived herself as a leader and being a candidate for an excellence award. However, on the other hand, her words describe additional aspects of coping and tension included in the initial encounter with the organization – moments which she describes as war (“I had to fight”). Particularly when she finds herself in the position of being the sole Arab woman, thus becoming emblematic as a token representation (Farrell and Barao, 2023). Such an encounter also includes excluding elements, as well as an inherent experience of coping with racism. Dealing with racism represents a significant hurdle in enhancing police diversity, as institutionalized racism may lead minority officers to feel like outsiders (Todaka et al., 2018). One of the issues which came up later in Lubna’s interview, and often in the other interviews, illustrating more than anything else the identity-related conflicts FAPO deal with from the beginning, is related to the fact that Police Academy candidates are sometime sent at a moment’s notice to assist the security forces in Jerusalem in routine policing action or unusual security-related events, which mostly develop in Eastern Jerusalem where Palestinians who are not Israeli citizens live. The confrontations between the security forces and the Palestinian population often take place around the El-Aktza Mosque (situated on the Temple Mount in Jerusalem, and perceived as a symbol of the struggle against Israel, due to its control of the city, beyond its religious status and holiness for all Muslims). This complexity is described by Salam a Muslim woman:

*The Temple Mount, I’m telling you, was a nightmare, a nightmare, a nightmare… and as far as the police is concerned, you need to assist, do your watch…*

Salam describes the difficult experience (“*a nightmare*”) and how in their day-to-day service, FAPO frequently make compromises, as they have to deal with conflicting values simultaneously rather than separately or sequentially (e.g., Boltanski and Thévenot, 2000; Oldenhof et al., 2014).

Similarly, Ruwan also related to these Jerusalem tasks, illustrating the conflictual identity-related experience involved in them:

*The assistance tasks in Jerusalem are one of the unpleasant things. It’s like standing there and seeing things that you as a Muslim do not want to see. It does not do you good but there’s nothing you can do about it. I’m not saying that there aren’t any good police officers, it’s just the opposite – the officers are good and humane and treat the people who have come to pray very well. But you never know how things will go, so… it’s not a simple situation at all… Going into El-Aktza with your hair untied [according to Muslim belief], this is a holy place where I have to wear a head scarf.. I did not know, I did not dream, I did not expect this, and still, because I want to be a police officer, I said God will forgive me, God knows what is in my heart and I will go in and I went in there. I did wear my helmet most of the time…but it’s not the same.* (single, Muslim).

Despite the fact that Ruwan discusses the police activity as mostly attempts to take into account the minority population, we understand that she had undergone a personally difficult experience regarding the crossing of the ethno-national-religious boundaries. Throughout the interviews, the FAPO describe positive service experiences and feelings of contributing to their origin communities, and in particular their role in relation to youth or to Arab women victims of domestic violence who, due to their presence, started to seek safety by turning to the police. That is, in these cases they could perceive their workspace as more positive, and they emphasize their gender identity, thus succeeding in dimming the ethno-national identity contradiction. There is no doubt that the situation experienced in the course, despite being temporary, is extreme in its identity-related complexity. Thus, Ruwan, who comes from a secular background, who had worked in ethno-national mixed places prior to joining the police, and expresses her satisfaction with her professional choice in many places in the interview, still emphasizes the earth-shattering experience she had undergone during the course – coping with the conflictual presence of the multiplicity of identities and the contradiction between her religious-civil-national identities and the demands of her professional identity.

### The problem with no name: “All this conflict”

Sa'ar (2023) claims that since the 1990s, the division between inclusion and exclusion of the Arab citizens in Israel has been growing more and more extreme. The FAPO, belonging to the Arab minority, suffer from different aspects of exclusion on the one hand, and their professional integration is part of the developing economic citizenship offered to them on the other. This identity wishes to avoid challenging Israel’s national-racist-exclusive character. The division between inclusion and exclusion – belonging vs. non-belonging (e.g., Anthias, 2008, 2020), and the matching hyphenated identity, are present in many interviews, but the officers express it vaguely, in a manner which can be named “the problem with no name.” For example, Amna does not call the national conflict by its name, rather referring to it vaguely:

*I do not think I have a problem with **‘that’** [the issue of national identity]. I do my job, at the end of the day I live in Israel, I do what I have to do. I know my job, I know my identity, and there’s nothing I can do [chuckle].* (34, married+2, Christian).

Amna explains the differentiation she manages to make between her professional-civilian identity and her ethno-national identity, in an almost disconnected manner. Likewise, Rasha says:

*I try to go into ‘**this’** as little as possible [why I am a police officer], because the minute I go there I enter a sort of mixed identity situation. Who am I, what am I, where have I come from, where did I go? I try to look forward as much as possible - know why I am here, what my goal is, what my ambition is, and believe what I want to believe. Follow my principles. I always remind myself why I joined the police force, what my goal is…* (29, single, Muslim).

Rasha, like others, relates to the identity-related complexity she is experiencing based on the national conflict using the impersonal term “this.” She makes it clear that it is impossible to ignore the multiple identities, or as she experiences and defines it, the “mixed identities.” However, she explains that in order to function as a police officer she must ignore the identity-related space which includes a structured identity contradiction, and focus on the other aspects which made her enlist. Similarly, Boltanski and Thévenot (2000, p. 213) claim that in order to deal with conflicting values when performing justification work people “extract themselves from the immediate situation and rise to a level of generality.” As such, people can be endowed with different values, being women, citizens, or employees. Ruwan uses a similar description when discussing her coping with the multiple identities she is experiencing:

*You know, joining the police force is not easy. Really not easy… because of the issues of credibility and loyalty. Are you loyal to the police and are you loyal to the state **and all this conflict** of Israeli Arabs, yes Palestine, yes no I do not know what… …and the point of view of people working with you, you always feel like you are somehow being examined…* (single, Muslim).

At the beginning, Ruwan opens with a direct reference to the identity complexity when she presents her identity dilemma through the question of loyalty. Indeed, she describes the situation as an encounter among different identity representations. In theory, the presence of minority police officers improves the accessibility of the police for minority citizens and communities. However, at an everyday level, this can introduce personal complexity for the FAPO themselves, being members of the same oppressed group (Todaka et al., 2018; Hasisi and Litmanovitz, 2021). Moreover, due to the small number of FAPO, Ruwan describes her daily experience as ‘tokenism’. She explains that she feels like she has become a representative symbol (“*you are being tested*”). However, although she initially describes the situation directly, later she talks about it as an amorphous conflict (“*this*”). It is important to remember that the interviewees did not mention the identity conflict as the main topic in the interview, referring to it indirectly or in response to my request as the interviewer. For example, it came up through reference to the issue of wearing the uniform.

### Wearing a uniform as a marker for boundaries-crossing

The uniform symbolizes an individual’s commitment to group norms and standardized roles, indicating a mastery of essential group skills and values. Yet, employees’ response to wearing a mandated uniform upon recruitment provides insights into various perspectives on its significance and its representation of a network of identity issues (Joseph, 1972; Pratt and Rafaeli, 1997). The way in which the uniform is used as a marker for the gradual process that the FAPO experience in front of their community is reflected in Hanan’s words:

*…And one day I really had to go home in uniform… and they began to understand that I’m really a police officer.* (Hanan, Christian).

One of the barriers for women in the police force is related to the appearance requirements (e.g., uniforms, hairstyles). The Israeli police has appearance requirements, but there are no rules for female officers wearing their hair short. Wearing uniforms in law enforcement agencies symbolizes organizational belonging, good citizenship and nationalism. The uniforms are part of the ways of asserting control, enforcing the corporate identity by creating a standardization of appearance and behavior, as well as helping to build a hierarchy. It is believed that a presence in uniform may strengthen the public’s trust in the police (Pratt and Rafaeli, 1997; Proctor, 1998; Stanko et al., 2012). However, wearing Israeli police uniforms for FAPO can become a non-verbal marker and emphasize the contradiction of their multiple identities. Similarly, Pratt and Rafaeli (1997) claim that the discussion of uniform includes complex notions of multilayered social identities. Uniforms serve as a vehicle for representing and negotiating a web of multiple and contradictory identity-related issues. Their study illustrates how an examination of organizational symbolism can offer a view into organizational identity and ambivalence, as well as identity conflicts. Indeed, in many interviews the uniform issue was raised as a symbol of the daily complexity the female officers cope with in the transition between societies. For example, Abir, a Druze woman, describes the duality she experienced through the spatial division she constructed for herself regarding the uniform, understanding the symbolic meaning of belonging the uniform represents:

*From the beginning I wore the uniform, but at the beginning before I entered the village, I would take off the uniform shirt and keep on the white t-shirt. Not so today, today I do not care. If I’m wearing uniform, I go home in uniform, I exit the car in uniform, I do not care anyone. In the beginning, because of my parents [who opposed her joining the police force], now when I see there’s support [of being a police officer] I do not care, I do not care anymore*.

According to Pratt and Rafaeli (1997), a seemingly simple symbol, such as an organizational uniform, may reveal the complex notion of social identity, comprising multiple layers of meaning. Wearing the uniform means public affirmation of their professional choice in a manner which cannot be limited only to people known as supporting this choice. Going about in uniform is a type of declaration which includes having a voice in ‘the world’ vis-à-vis the common socio-national voices. However, as FAPO who have to perform justification work as a coping mechanism almost daily, they have to adopt a particular course of action, notably in cases when their opinions or actions are challenged by others (Oldenhof et al., 2014). Abir’s words demonstrate the gradual process she had undergone, carrying on a dialog with the objecting voices around her. Support of the nuclear family was pointed at in the interviews as the default situation for serving in the police force, but sometime it is not sufficient. Belonging to a minority group and a collectivist society, the community circles still serve as a central social element for them, and therefore an additional element mentioned in the interviews was different opposition patterns by the community (Meler and Benjamin, 2023).

Not wearing uniforms in their residential areas was a way of keeping their employment a secret, complying with what Wasserman and Frenkel (2020, p. 1610) call “a matrix of contradicting visibility regimes.” Exposure to the community, as Abir describes, is done gradually and takes time, allowing for a personal internal process the female officer undergoes. The strong meaning of moving between identities which takes place while crossing spatial boundaries in the context of Israeli segregationist politics can also be seen in Rasha’s words:

*In the beginning I was half and half. I could not make up my mind. And after eight months I put on the uniform.* (29, single, Muslim).

Rasha’s words also demonstrate a gradual process symbolizing movement among identities, boundary crossing and a negotiation the female officers may carry on among their different identities. This process begins hesitatingly, as Rasha describes, with the wearing of the uniform serving as a symbol of deeper identity-related experiences, ending with the act of making her profession clear. The hesitation Rasha describes regarding the uniform demonstrates its deep level and the willingness to move among identities. Thus, for example, Rola, who was not wearing a uniform during the interview, replies quite clearly when asked about it: *“No, no! To make a point! I do not want to wear a uniform, not to stand out too much.”* Different departments of the police force, such as Youth, Intelligence and Investigations, allow both Jewish and FAPO female officers to wear civilian clothing. However, Rola’s words also reflect the meaning of this custom and the fact that working out of uniform is a possibility and definitely an advantage for them.

Not wearing uniforms can signify negotiations over identity, which can be intentional. Such deviations from a dress code enable individuals to push the boundaries of their group’s norms, as the symbols are tied to the organization’s deeply embedded values and ideals, as well as its social structure (Stanko et al., 2012). Similarly, Nariman also related to the uniform issue as a conflict concerning civic and ethno-national identity:

*When I am at work, there is no way I am taking off my uniform, or when I am coming home – because I have nothing to fear. I am a police officer – I must wear my uniform.. I represent the police, I represent the state, I must not be afraid. As I protect civilians and the police, the police must protect me. This is my personal view. If I have errands to run after work, I wear my uniform and I am not afraid, but when I am on vacation, I prefer not to stand out or show that I am a police officer*. (28, single, Christian).

Nariman makes the direct connection by her very existence in uniform: *“I represent the police, I represent the state,”* and although it is clear that she is referring directly to the identity-related complexity which is part of her joining the police force, it is also clear from her words that she feels good about her decision and proud of being a police officer, seeing it as a meaningful mission *“As I protect civilians.”* However, her words also show the difficulty inherent in crossing boundaries. The practice of putting on and taking off the uniform allows the officers to move about and carry on a negotiation among the different identities; and beyond the dress issue, this shows that social identities in organizations can be multi-layered (e.g., Pratt and Rafaeli, 1997). This might seem like spatial or functional movement (between the home and professional space), but their words demonstrate that the spatial divisions have a deeper meaning, symbolizing belonging. Despite having made peace with their joining the police force, they describe how they explicitly deal with the uniform issue (where to wear it and where not), and in a deeper sense when they can emphasize their professional identity, which in the case of FAPO means they are crossing boundaries and moving from an ethno-national identity to acceptance of a civil identity. Due to Israel’s complex conflictual social structure, the divisions are not limited to movement among Jewish spaces or mixed cities, but also mark boundaries of inter-social belonging. For example, several officers mention the difference in their religious identity as allowing for different levels of belonging. Thus, for example, some mention the differences between towns or villages with a Christian or Muslim majority. This can be glimpsed between the lines in Alaa’s words, as she explains that despite her being a Christian, and her family and community exhibiting less resistance to her joining the police force, she still needed to deal with potential opposition in her neighborhood:

*I come from a Muslim neighborhood with only three Christian houses, which is important. Leaving this neighborhood in uniform, everyone looking at me like this, but I do not care, I come home and leave in uniform, it’s OK*. (32, married+2, Christian).

However, it is not only religious affiliation which characterizes the female officers’ coping, but also the level of nationality and political involvement of the specific town or village. Crossing boundaries is not an experience they encounter only in their direct circles of belonging – family, community, and town/village, but also in the public sphere in the town/village where they live. From Dalal’s words we gather that to a certain extent it is possible to claim that the uniform may serve as a litmus paper for examining the opposition to their joining the police force:

*In some Arab towns/villages there’s hatred toward police officers. But here [an Arab village where she is serving today] I have not encountered this… here there’s no problem going about in uniform. I walk about on foot, I go for a run… yesterday I ran in the middle of the village* (28, married+1, Muslim).

The virtual space also presents a source of opposition, and thus a space of hostility and danger. Indeed, most officers relate to the fact that due to the social and community-based opposition they experience regarding their joining the police force (Meler and Benjamin, 2023), they avoid any exposure on social media and make sure they are not tagged in photos which may identify them as police officers. Thus, Rawaa explains how she ensures limited visibility:

*I do not discuss politics with just anyone, unless I know them, because I do not think I should open doors which I’m not sure I’ll be able to close later. I also do not upload photos with me in uniform on social media. I do not need people’s reactions… It’s enough that I do what I love…* (27, single, Muslim).

Rawaa’s words and the safety measures she takes were repeated by many female officers. However, May expressed a different, unique, voice:

*In the course I began to wear uniform and go about wearing it… and generally, I always have my pistol with me, even when wearing civilian clothes… Everyone in the village knows I am a police officer; they know… it goes around by word of mouth. They know… if people talk, I do not pay any attention, I love my job and I do not care. And when I’m in uniform I sometimes post on Instagram, you know, in uniform… I’ve received some responses, like one or two responses were shocked that I had enlisted. I did not pay any attention; I should not have to explain myself. People who know me, friends and such, they will not cancel me now because I’ve enlisted…* (25, single, Muslim).

Hamdan-Saliba and Fenster (2012) argue that for women, crossing boundaries offers new meanings and references. Crossing boundaries, which is part of the effort to improve employment opportunities, becomes in a deeper way a practice challenging their ‘hollow citizenship’ (Jamal, 2007). While May says that she feels she has no need to explain, expecting that the fact that she is a police officer will not raise much oppositional responses, other female officers presented different rhetoric, attempting to justify their conflictual identity situation.

### Bargaining with a hybrid identity

The words of the FAPO thus far, expressing their experience of identity complexity, demonstrate what emerges in the wider study - that recruitment into the Israeli police force is an action in a space where their presence as FAPO may be considered as cooperation with the majority, the hegemonic group, and in some circles as betrayal. Therefore, in order to hold their position and maintain the opportunity for a ‘quality job’, they must position themselves in a way that refutes the conflicting identity experiences.

*I say this… OK, you grew up on a history of Palestine and everything is yours, that’s your right. Everyone has a right to believe what they believe, this is what you believe, or you. It’s your right, but not to come and tell us – no, do not enlist… I believe in something; you believe in something else. Like you believe in a religion, for example Islam, I believe in Christianity. Every person has his/her belief. It’s your right, it’s a democratic country, you can believe in whatever you want to… but so can I…* (*May*, 25, single, Muslim).

In her interview, May relates to the identity-related issue as if she is addressing an imaginary audience, responding directly to the opposition often directed at the female officers by elements in the community using arguments emphasizing the contradiction in national identity. She presents the identity-related contradiction as part of a legitimate discourse of pluralism and multiple opinions taking place in a democracy. Furthermore, she continues by defining the objections as a form of faith that distances itself from majority-minority relations, as well as from all the national conflict and civil inequality existing in the State of Israel. In doing so, it seems that the conceptualization she uses becomes a resource allowing her to enlist and position herself as more pluralistic or tolerant than the rest of her community.

Other female officers wish to emphasize a different identity-related aspect of their cluster of identities. Thus, for example, Miriam a Christian woman, explains:

*We were born Israelis, we are Israelis and we have to obey the laws here, this is the state we live in and we have to give and contribute whatever we can. Even if I do not serve in the army but there’s something else I can contribute, I will do so… I will give what I can.*

It seems that Miriam is relating in her arguments to the republican citizenship discourse (Peled, 2008). This discourse does exist in Israel, but only regarding Jewish citizens, demanding that they contribute to the country, and at the same time excluding the Arab citizens. In the context of the fact that Israel differentiates between its Jewish and Arab citizens regarding their civilian obligations and rights, Miriam seems to ignore this segregationist reality, emphasizing the civil obligation of contribution to the state. While Miriam emphasizes her civil identity, other female officers emphasize their religious identity over their national one. Thus, for example, Gadir provides a negative answer to my question regarding whether or not she feels the complexity of being a female police officer re-her national identity:

*No, for the Christians this is not a problem, less so. But other groups, sometimes suspects sit in front of me and wonder, like, why am I doing this job? And I reply that it’s a great job and a great mission… for society, for women…* (31, single, Christian).

A softening of the contradiction issue related to national identity was evident in Gadir’s words, when she directed the discussion to the religious identity as creating an internal difference among Arab citizens in Israel. Gadir’s rhetoric, constructing inter-ethnic differentiations, echoes Israel’s official policy which often uses ‘divide and conquer’ among different religious communities and Israel’s Arab citizens. In addition, Gadir emphasizes the importance of the professional side and her contribution to society, particularly for women, in a manner providing her with a sense of worth which overshadows all difficulties (Meler and Benjamin, 2023). In a similar manner, Alaa also refuses to be perceived as representing the collective:

*And they say [about me] that she is an embarrassment to the Palestinian people. Sorry, I do not represent the Palestinian people…I do not represent them. I am **myself**. I have not come here to represent a community, a religion, a nationality, a population, no. I’ve come as Alaa, only me* (44, divorced+2, Christian).

Alaa’s words resonate against the background of the conflictual reality in Israel, where as a minority woman that has crossed boundaries, she feels that there are members of her community that objected to her breaching the ethno-national boundary and working in what is perceived as an embarrassment to the Palestinian people. Despite acknowledging social expectations (refraining from accepting a job in an organization that represents the dominant majority), she frees herself from belonging to the community (e.g., Anthias, 2008, 2020) when she chooses to enlist in the police-a job that is considered less normative for Arab women.

Another significance unveiled through the crossing of ethno-national boundaries is tied to the fact that the State of Israel was formed around Jewish nationalism, and this interest is expressed in the definition of Israel – a ‘Jewish and democratic’ state. This nature of the state has clear practical manifestations – state symbols are drawn from Jewish sources (anthem and flag), the days of rest and the days of remembrance correspond to the symbols and the Hebrew calendar, and the Hebrew language enjoys official superiority. The public image of public life is based on Jewish culture (Smooha, 2002, 2006).

The assimilation of Jewish religious symbols into public spaces and the lack of a common civil ‘Israeliness’ creates a sense of alienation and exclusion for Arab women within these spaces. Consequently, during their service, FAPO might encounter contradictory situations, being expected to pledge allegiance to the flag or sing the Israeli national anthem, symbols not universally shared among all citizens of the country. This is especially reflected in ceremonies, which have a symbolic status based on the assumption that the participants associate themselves with the organization and the state. For example, Ruwan replies to a question regarding the national anthem:

*Q: I understand that there are certain ceremonies which you have to attend. How do you manage there, for example with singing the anthem?*

*A: As usual. As usual. What’s the problem? It’s my country, is not it? I stand up, what I did in the course, at least with myself, I respect the people on both sides. Arabs also died because of the state, the same thing, so I respect them, salute them, salute the flag. After all I’m an Israeli, that’s all there is to it, I have an Israeli ID card, I have an Israeli passport. It does not matter even if people say to me you are an Arab, but I’m Israeli, it does not matter.* (single, Muslim).

Israel’s national anthem does not represent the national feelings of its Arab citizens. Its lyrics reflect Zionist ideology, and therefore non-Jewish Israelis holding public jobs often relate to their difficulty in articulating the national anthem’s words. Some choose to respect the situation by standing up, but do not sing it. Ruan emphasizes her civic belonging, describing a very rational reality, very different from the tension-filled Israeli reality of the past few years. Thus, she draws a symmetrical, harmonic picture which does not characterize Israeli reality, as if she simulating a type of “civic nationalism built by a community of equal, rights-bearing individuals who are united in patriotic attachment to a shared set of political practices and values” (Ignatieff, 1993, p. 6).

Alaa’s words also describe a simple, compromising solution:

*At the swearing-in ceremony I was asked: “Would you like to swear on the New Testament or the Bible?” I said the Bible is fine, we also believe in the Bible. Christianity does not exist without the Bible, so… I swore on the Bible.* (44, divorced+2, Christian).

Lubna also discusses the swearing-in ceremony at the end of the course:

*At the end-of-course ceremony I felt proud and it was very moving, there are no words to describe my mood, my parents and my commanding officers who accompanied me to the ceremony, everything was perfect, very moving… and we sang the anthem but I did not hold the flag* (29, single, Muslim).

Lubna emphasizes the excitement she and her family felt at the end-of-course ceremony. The fact that the national anthem was played at the ceremony did not detract from this excitement. However, while it is informally accepted in civil organizations that non-Jewish citizens should respect the anthem, stand when it is sung or salute if required, but are not obligated to sing, holding the national flag may be experienced, as Lubna’s words suggest, as a more resonant and visible action. These strategies, described by the officers, demonstrate a negotiation they carry out with the identity cluster stemming from their crossed-over placement. Sometimes they feel their professional identity and sometimes their civic identity, where they emphasize their belonging to the state by relating to the civic discourse. By emphasizing these identities, they overshadow other identity-related elements which may present a conflict. However, the fact that the participants have managed to integrate into the organization, and express satisfaction, is not related only to the professional aspect and achieving the goal of a high-quality job, or due to their social-civic contribution to their communities. As minority women, belonging to the geographic and social periphery, it seems that serving in the police force strengthens for many of them an experience of recognition and belonging.

## Discussion and conclusion

During the last decades, the demographic characteristics of police department personnel worldwide have changed, but it is still rare to find a department that mirrors the population it serves in terms of gender and race/ethnicity (Farrell and Barao, 2023). In many police organizations around the world, much effort is spent on increasing diversity. This aim stems from a management perspective, arguing that strengthening representative bureaucracy would allow various populations to experience service providers as identified with them nationally and religiously, and establish trust in the social service (Todaka et al., 2018).

The Israeli police force offers an employment path to young Arab women, aiming to strengthen gender and ethno-national diversity in its ranks. In recent years, dozens of Arab women have accepted this occupational path. As minority women, they face ongoing discrimination, underdevelopment of their residential areas and a narrow employment opportunity structure, as well as a prevalence of traditional gender ideologies and control of women’s sexuality, leaving many of them in a precarious position. Therefore, they yearn for opportunities that will enable them to rely heavily on public sector service positions (Abu-Rabia-Queder, 2017; Meler and Benjamin, 2022; Sa'ar, 2023). Following Anthias (2008, 2020) conceptualization, this can be seen as expanding employment opportunities by crossing ethno-national boundaries. The police force offers them a path to a quality job with economic independence and a sense of satisfaction, but following this path involves crossing ethno-national and gender boundaries, which poses many dilemmas for them.

There is a consensus concerning the centrality of ethno-nationality as the principle governing social life in Israel, leaving non-Jewish groups excluded from major arenas and alienated from the core values of Israeli society (Shafir and Peled, 2002; Sa'ar, 2023). Smooha (2020) Index studies show that both the Arab and the Jewish public has undergone an exacerbation of attitudes and some radicalization in the last decade.

Concerning the issue of ethno-national identity, the FAPO experience different levels of objections and even hostile reactions from family members, the community, and citizens detained for questioning (Meler and Benjamin, 2023). However, in the present article I focus on the ethno-national boundaries crossing among FAPO in Israel from their subjective perspective. Examination of the meaning of joining the police force from the female officers’ point of view contributes to the anchoring of minority female police officers’ experiences within the specific familial, cultural, spatial and political context from which they leave for work and in which they experience the police force track offered to them, while emphasizing the ethno-national identity complexity involved in serving in this force. As FAPO, they find themselves in a multi-axis liminal place – between the civilian, the ethno-national and the historical. Within the Israeli civil arena, the Arab citizens are positioned in the liminal space existing between ethno-nationality, citizenship and state. It is possible to claim that the officers wish to cope with the liminality from within this discourse and against it (e.g., Fakhoury, 2021). Anthias (2008, 2020) social processes are manifested in this experience of minority female police officers who insist on their civil belonging and manage to cross ethno-national-spatial-gender boundaries, improving their chances of integrating into a quality job.

However, the present article further illuminates their subjective experiences and the coping mechanisms they use to perform justification work (Boltanski and Thévenot, 2000; Oldenhof et al., 2014) in order to deal with conflicting values. The findings show that FAPO make compromises, only vaguely refer to the national conflict, focus on their professional identity at a level of generality, resort to the discourse of civil nationalism (Ignatieff, 1993), and practically speaking, learn to move among identities when they are out of uniform or avoid emphasizing their presence in conflictual spaces (political discussions or virtual spaces). Their performing of justification work entails considerable work, constantly maintaining and recrafting (e.g., Oldenhof et al., 2014).

The contribution of the present study lies in the in-depth understanding of the interpersonal ethno-national processes associated with the experiences of minority female police officers in their own land (not immigrants), part of an ‘involuntary minority’ (Ogbu, 1990), showing how they develop, adopt and retain multiple identities, crossing ethno-national boundaries. While Ogbu (1990) claims that the status of ‘involuntary minorities’ increases the feeling of non-belonging and creates a negative attitude toward the system identified with the majority group, the current study describes a spectrum of voices serving as part of a rhetoric bargaining with the identity complexity. The findings show that female officers integrate a type of national work into their service (‘doing nationality’), when they have to challenge their identity due to their professional connection to the state and its institutions, as part of their status of belonging to a minority group, and in a manner emphasizing their national identity.

To a certain extent it can be argued that they see the concept of civic nationalism characteristic of culturally developed nations that can, from a position of self-confidence, approach each other on an equal footing, seeking cooperation on the basis of mutual respect (Tamir, 2019) as a form of coping mechanism, because despite the identity-related contradictions inherent in the situation of FAPO in Israel, due to their continuous justification work they manage to emphasize alternative discourses (gender-related, religious, civil) which justify their service and blur the complexity.

The limitation of the study is its concentration on the FAPO perspective regarding ethno-national boundary-crossing. A broader, unaddressed topic, includes gender boundary-crossing and the conflict with gender expectations imposed on women, especially in more conservative societies. Additionally, as the study is a short-term one, the limitations are clear and the perspective focused on a specific point in time does not allow to examine the meaning of crossing the ethno-national boundary over time in light of the fact that specific political developments make this either easier or more difficult. It is safe to assume that the officers’ subjective experiences may change as ethno-national tensions become worse.

Similarly, it can be argued that the political reality in Israel is currently subject to changes and instability, and anti-democratic measures and attitudes have been aggravated in the past year. It can therefore be assumed that the labeling process of the Arabs as illegitimate and stopping investments and improvement of living conditions in the Arab sector may also exacerbate Arab citizens’ attitudes. When political changes occur (if at all), this will have implications on the diversity policy in governmental organizations. This, of course, may also have consequences for the subjective experience of FAPO, which was presented in the current article as complex, and beyond the scope of the Israeli case study.

The present article wishes to provide a voice and visibility to the experience of minority women crossing ethno-national boundaries and integrating into the general labor market against the many barriers they experience both from the state and from the society to which they belong, and in light of the growing understanding of the importance of their crossing the boundaries of their ethnic enclaves in which they suffer from a paucity of work opportunity and integration into the public sector. Analysis of the identity complexity of Arab women who chose to work as police officers adds a destabilizing step, albeit a modest one, to the perception emphasizing uniformity and universality of social and cultural voices regarding their influence on women in general and minority women specifically. In the present era, the prevailing assumption is that a more representative police department will have a greater chance to represent the community’s interests, as well as help shift the police culture in breaking down racial prejudices by gaining knowledge and increasing their understanding of different cultures (Todaka et al., 2018). The issue raised in the current article cannot be ignored, and is a layer of the diversity policy. As shown in the present study, it has a contribution due to the fact that scholars and police departments need to consider how police officers experience their service (a ‘craft knowledge’ from their subjective perceptions and interpretations), while working in organizations focused on increasing gender and racial/ethnic diversity (e.g., Farrell and Barao, 2023), but at the same time are also committed to different family and community circles of belonging. These insights illuminate the organizational knowledge regarding minority policing and diversity policy taking within a deeply divided society along ethnic lines characterized by tense relations between the minority and the police.

## Data availability statement

The raw data supporting the conclusions of this article will be made available by the authors, without undue reservation.

## Ethics statement

The studies involving humans were approved by Zefat Academic College Ethics Committee. The studies were conducted in accordance with the local legislation and institutional requirements. The participants provided their written informed consent to participate in this study.

## Author contributions

TM: Writing – original draft.

## Footnotes

^1^The research participants are all Israeli citizens and identify themselves as Arabs and not as Palestinian-Arabs, therefore to indicate their identity I use the term “Arab women in Israel” or “The Arab society” as they expressed themselves, and not as I have done in other studies (Meler, 2019, 2021; Meler and Benjamin, 2023) where the participants defined their identity as Palestinian-Arabs or Arab-Palestinian.

^2^A broader aspect, not covered in this article, relates to Arab women’s crossing of gender boundaries. The perception of police forces as masculine professions highlights the contradictions between their professional identity and the gender expectations placed on women, particularly in more conservative societies.

^3^The integration of FAPO into the Israel Police is a phenomenon that has intensified in the last 5 years. From the authorities’ point of view, along with understanding of the study’s importance, concerns may have arisen due to the sensitivity involved in such research and the need to protect the interviewees.

## References

[ref1] Abdo N. Lentin R. (2002). Women and the politics of military confrontation: Palestinian and Israeli gendered narratives of dislocation. Berghahn: Berghahn publications.

[ref2] Abu-Rabia-Queder S. (2017). The emergence of a class identity: Naqab Palestinian professional women. Israel: Magnes, Hebrew.

[ref3] Acker J. (1990). Hierarchies, jobs, bodies: a theory of gendered organizations. Gend. Soc. 4, 139–158. doi: 10.1177/089124390004002002

[ref4] Anthias F. (2008). Thinking through the lens of translocational positionality: an intersectionality frame for understanding identity and belonging. Translocations: Migration and Social Change 4, 5–20.

[ref5] Anthias F. (2020). Translocational belongings: Intersectional dilemmas and social inequalities. UK: Routledge.

[ref6] Arweck E. Nesbitt E. (2010). Close encounters? The intersection of faith and ethnicity in mixed faith families. J. Beliefs Values 31, 39–52. doi: 10.1080/13617671003666696

[ref7] Bauml Y. (2010). “Sixty-one years of supervised abandonment: decoding Israeli governmental policy toward Arab citizens” in Abandoning state supervising state: Social policy in Israel. eds. Katz H. Tzfadia E. (Israel: Resling [Hebrew]), 35–54.

[ref8] Beijaard D. Meijer P. C. Verloop N. (2004). Reconsidering research on teachers’ professional identity. Teach. Teach. Educ. 20, 107–128. doi: 10.1016/j.tate.2003.07.001

[ref9] Ben-Asher S. Gottlieb E. E. Alsraiha K. (2023). Multiple identities: young Bedouin professionals challenging their socio-cultural representations. Social Identities. 28, 747–765. doi: 10.1080/13504630.2023.2166031

[ref10] Boltanski L. Thévenot L. (2000). The reality of moral expectations: a sociology of situated judgment. Philos. Explor. 3, 208–231. doi: 10.1080/13869790008523332

[ref11] Bradbury M. Kellough J. E. (2011). Representative bureaucracy: assessing the evidence on active representation. Am. Rev. Public Adm. 41, 157–167. doi: 10.1177/0275074010367823

[ref12] Brewer B. M. (1991). The social self: on being the same and different at the same time. Personal. Soc. Psychol. Bull. 17, 475–482. doi: 10.1177/0146167291175001

[ref13] Bruner J. S. (1990). Acts of meaning. US: Harvard University Press.

[ref14] Charmaz K. (2015). “Grounded theory” in Qualitative psychology: A practical guide to research methods. ed. Smith J. A. (US: SAGE), 53–85.

[ref15] Dhont K. Cornelis I. Van Hiel A. (2010). Interracial public–police contact: relationships with police officers’ racial and work-related attitudes and behavior. Int. J. Intercult. Relat. 34, 551–560. doi: 10.1016/j.ijintrel.2010.07.004

[ref16] Edwards R. (1993). “An education in interviewing: placing the researcher and the research” in Researching sensitive topics. eds. Renzetti C. M. Lee R. M. (US: Sage), 181–196.

[ref17] Fakhoury A. (2021). Revisiting the question of the 'Israeli Arab': From the politics of suspicion to the politics of identity and morality. The Forum for Regional Thinking, Van Leer Institute. Retrieved from https://www.regthink.org/reviewing-the-israeli-arab/

[ref18] Farrell C. Barao L. (2023). Police officer perceptions of diversity efforts: a disconnect between the goals and the methods. Police Pract. Res. 24, 216–231. doi: 10.1080/15614263.2022.2098127

[ref19] Gharrah R. (2018). Arab society in Israel: Population, society, economy (9). Van Leer Institute, Hakibbutz Hameuchad. US.

[ref20] Granit E. (2021). Young people in Israel 2021: Data review. The Ministry of Social Equality. Retrieved from https://www.gov.il/BlobFolder/reports/israel_youth_data_2021/he/israel_youth_data_2021.pdf

[ref21] Green E. (2021). Examining the experiences of women police leaders in Illinois. Illinois Criminal Justice Information Authority. Retrieved from https://icjia.illinois.gov/researchhub/articles/examining-the-experiences-of-women-police-leaders-in-illinois

[ref22] Hamdan-Saliba H. Fenster T. (2012). Tactics and strategies of power: the construction of spaces of belonging for Palestinian women in Jaffa–Tel Aviv. Women's Stud. Int. Forum 35, 203–213. doi: 10.1016/j.wsif.2012.03.022

[ref23] Hasisi B. Litmanovitz Y. (2021). Governability and effectiveness in policing divided societies: police commanders' perspective of Arab society in Israel law and policing. Law Society & Culture 4, 265–304.

[ref24] Hasisi B. Weitzer R. (2007). Police relations with Arabs and jews in Israel. British J. Criminol. 47, 728–745. doi: 10.1093/bjc/azm027

[ref25] Hinton M. S. (2006). The state on the streets: Police and the politics in Argentina and Brazil. US: Lynne Rienner Publishers.

[ref26] Ignatieff M. (1993). Blood and belonging: Journeys into the new nationalism. Farrar, Straus and Giroux, US.

[ref27] Israel Central Bureau of Statistics. (2023). Israel's Independence Day 2023. Retrieved from https://www.cbs.gov.il/he/mediarelease/DocLib/2023/133/11_23_133b.pdf

[ref28] Jamal A. (2007). Nationalizing states and the constitution of ‘hollow citizenship’: Israel and its Palestinian citizens. Ethnopolitics 6, 471–493. doi: 10.1080/17449050701448647

[ref29] Jamal A. Almog-Bar M. Koukvine V. Eseed R. (2019). Arab Palestinian civil society organization in Israel. Israel: The Walter Lebach Institute for Jewish-Arab Coexistence Through Education Tel-Aviv University.

[ref30] Jamjoom L. A. Mills A. J. (2022). Narratives of workplace resistance: reframing Saudi women in leadership. Hum. Relat. 76, 1–35. doi: 10.1177/00187267221087593

[ref31] Joseph N. Alex N. (1972). The uniform: a sociological perspective. Am. J. Sociol. 77, 719–730.

[ref9002] Khattab N. Miaari S. (Eds.). (2013). Palestinians in the Israeli labor market: A multi-disciplinary approach. Springer.

[ref32] Lim H. H. (2006). Representative bureaucracy: rethinking substantive effects and active representation. Public Adm. Rev. 66, 193–204. doi: 10.1111/j.1540-6210.2006.00572.x

[ref33] Linnehan F. Konrad A. M. (1999). Diluting diversity: implications for intergroup inequality in organizations. J. Manag. Inq. 8, 399–414. doi: 10.1177/105649269984009

[ref9003] Meler T. (2017). The Palestinian family in Israel: Simultaneous trends. Marriage Fam. Rev. 54, 781–810.

[ref9004] Meler T. (2019). Underemployment vs. relocation: Coping mechanisms of Palestinian women in Israel with patriarchal and spatial impositions. Sociol. Spectr. 9, 300–318.

[ref9005] Meler T. (2021). In search of work: Israeli-Palestinian families migrating to Beersheba. Community Work Fam. 24, 392–414.

[ref9006] Meler T. Benjamin O. (2022). Transitions in the meaning of belonging: The struggle for enhanced access to resources among Palestinian-Arab women in Israel. Ethn. Racial Stud. 45, 1693–1714.

[ref9007] Meler T. Benjamin O. (2023). Research report: Policewomen and leaders: Dimensions of gender equality in the integration of Arab-Palestinian policewomen in the Israel Police. The Gender Forum, Law and Social Policy, The faculty of Law, Haifa University. [Hebrew]. Retrieved from https://drive.google.com/file/d/1qXtlQZ5W5p9-PH79EYqtYhnDFifezG19/view

[ref34] Miaari S. Hadad Haj-Yahya N. (2017). NEET among young Arabs in Israel. Center for Democratic Values and Institutions. Retrieved from https://en.idi.org.il/media/9319/neet-among-young-arabs-in-israel.pdf

[ref35] Ogbu J. U. (1990). Minority education in comparative perspective. J. Negro Educ. 59, 45–57. doi: 10.2307/2295291

[ref36] Oldenhof L. Postma J. Putters K. (2014). On justification work: how compromising enables public managers to deal with conflicting values. Public Adm. Rev. 74, 52–63. doi: 10.1111/puar.12153

[ref37] Peled Y. (2008). The evolution of Israeli citizenship: an overview. Citizsh. Stud. 12, 335–345. doi: 10.1080/13621020802015487

[ref38] Popper-Giveon A. Keshet Y. (2020). Regardless of religion and race: Ethno-national tensions between jews and Arabs in the Israeli health system. Israel: Carmel.

[ref39] Pratt M. G. Rafaeli A. (1997). Organizational dress as a symbol of multilayered social identities. Acad. Manag. J. 40, 862–898.

[ref40] Proctor T. M. (1998). (Uni)forming youth: girl guides and boy scouts in Britain, 1908-39. Hist. Work. J. 45, 103–134. doi: 10.1093/hwj/1998.45.103

[ref41] Reinharz S. (1992). Feminist methods in social research. Oxford: Oxford University Press.

[ref42] Rudich-Cohen A. Sheperman T. K. (2015). Ultra-Orthodox youth and Arab youth: Review of the issue of young people in the Arab and Ultra-Orthodox sector in Israel 2015. Midot. Retrieved from http://www.midot.org.il/Sites/midot/content/File/%D7%93%D7%95%D7%97%D7%95%D7%AA/Midot%20-%20Arab%20and%20Orthodox%20Young%20Adults%20-%20Hebrew.pdf [Hebrew].

[ref43] Sa'ar A. (2023). Economic citizenship at the intersection of nation, class, and gender: The case of Palestinian women in Israel. Curr. Anthropol. 64. doi: 10.1086/723299

[ref44] Sa'ar A. Younis H. (2021). Diversity: Palestinian career women in Israel. UK: Open University Press.

[ref45] Shafir G. Peled Y. (2002). Being Israeli: The dynamics of multiple citizenship. Cambridge: Cambridge University Press.

[ref46] Smooha S. (2002). The model of ethnic democracy: Israel as a Jewish and democratic state. Nations and Nationalism 8, 475–503. doi: 10.1111/1469-8219.00062

[ref47] Smooha S. (2006). “Is Israel Western?” in Comparing modernities: Pluralism versus homogeneity. Essays in homage to Shmuel M. Eisenstadt. eds. Ben-Rafael E. Sternberg Y. (Netherlands: Brill Academic Publishers), 413–442.

[ref48] Smooha S. (2020). Still playing by the rules: Index of Arab-Jewish relations in Israel 2019. University of Haifa. Retrieved from https://drive.google.com/file/d/1whXPdn1p8HRX3XRoF-DkJJFXyKpvu77P/view

[ref49] Sodhi P. (2008). Bicultural identity formation of second-generation Indo-Canadians. Can. Ethn. Stud. 40, 187–199. doi: 10.1353/ces.2010.0005, PMID: 20734567

[ref50] Stanko B. J. Bradford J. B. Hohl K. (2012). A golden thread, a presence amongst uniforms, and a good deal of data: studying public confidence in the London metropolitan police. Polic. Soc. 22, 317–331. doi: 10.1080/10439463.2012.671825

[ref51] Tajfel H. Turner J. C. (1986). “The social identity theory of intergroup behavior” in Psychology of intergroup relations. eds. Worchel S. Austin L. W. (UK: Nelson-Hall), 7–24.

[ref52] Tamir Y. (2019). Not so civic: is there a difference between ethnic and civic nationalism? Annu. Rev. Polit. Sci. 22, 419–434. doi: 10.1146/annurev-polisci-022018-024059

[ref53] Todaka N. Huffb J. Jamesc L. (2018). Investigating perceptions of race and ethnic diversity among prospective police officers. Criminology and Criminal Justice Faculty Publications 19, 490–504. doi: 10.1080/15614263.2018.1428097

[ref54] Wasserman V. Frenkel M. (2020). The politics of (in)visibility displays: ultra-orthodox women maneuvering within and between visibility regimes. Hum. Relat. 73, 1609–1631. doi: 10.1177/0018726719879984

